# Paradoxical diurnal cortisol changes in neonates suggesting preservation of foetal adrenal rhythms

**DOI:** 10.1038/srep35553

**Published:** 2016-10-18

**Authors:** Masahiro Kinoshita, Sachiko Iwata, Hisayoshi Okamura, Mamoru Saikusa, Naoko Hara, Chihoko Urata, Yuko Araki, Osuke Iwata

**Affiliations:** 1 Department of Paediatrics and Child Health, Kurume University School of Medicine, Fukuoka, Japan; 2 Centre for Developmental and Cognitive Neuroscience, Kurume University School of Medicine, Fukuoka, Japan; 3 Faculty of Informatics, Shizuoka University, Shizuoka, Japan

## Abstract

Studies suggested the presence of foetal adrenal rhythms of cortisol, which are entrained in antiphase to maternal rhythms. In contrast, neonates are thought to have no adrenal rhythm until 2–3 months after birth. To test the hypothesis that a foetal-type adrenal rhythm is preserved after birth, saliva samples were collected from 65 preterm/term infants during hospital stay (30–40 weeks corrected age) at 10:00 and 19:00 h. Cortisol levels were assessed for their diurnal difference and dependence on antenatal/postnatal clinical variables. Cortisol levels were lower during periods 15–28 days and >28 days than ≤5 days of life. Lower cortisol was associated with pregnancy-induced hypertension (PIH), gestational age <28 weeks, and mechanical ventilation after birth. Higher cortisol was associated with vaginal delivery and non-invasive ventilation support at saliva collection. PIH and non-invasive mechanical ventilation at saliva collection were associated with cortisol levels even after adjustment for postnatal age. Cortisol levels were higher in the evening than in the morning, which was unassociated with gestational and postnatal age. Higher cortisol levels in the evening suggest the preservation of a foetal-type diurnal rhythm. Cortisol levels are associated with intrinsic and extrinsic variables, such as PIH, delivery mode, gestational age, and respiratory conditions.

Non-invasive salivary markers have extensively been used in clinical and psychological studies to assess the circadian rhythm and stress response involving children and adults^1^,^2^,^3^,^4^,^5^,^6^,^7^. In adults, there is a diurnal cortisol rhythm with its acrophase shortly after awaking. Previous studies in newborn infants have suggested no diurnal rhythm of salivary cortisol shortly after birth, whereas an adult-type circadian rhythm is observed 2–3 months after birth^8^,^9^,^10^,^11^,^12^,^13^. However, the mechanism of how a mature adrenal circadian rhythm is achieved remains largely unknown.

In contrast to the lack of an adrenal circadian rhythm in newborn infants, Serón-Ferré and colleagues reported that cortisol levels of cord blood immediately obtained after elective caesarean birth had a clear diurnal rhythm with the acrophase in the afternoon^14^. This finding suggested that foetuses have an adrenal circadian rhythm, which is entrained in antiphase to the maternal rhythm. Interestingly, our previous study, which involved collection of serial saliva samples of newborn infants every 3 h over 24 h, suggested the presence of a modest diurnal rhythm, with its acrophase late in the afternoon^15^. Additionally, we found the presence of an alternative acrophase, which was observed approximately 3 h after birth only during the first 5 days of life^15^. This finding suggested entrainment of the adrenal clock by activation of the hypothalamus–pituitary–adrenal (HPA) axis at birth.

Apart from diurnal rhythms, plasma cortisol levels increase towards the end of pregnancy, and this is further accelerated during delivery^16^. Studies in preterm infants suggested that, after birth, cortisol levels decline to a nadir over approximately 8 weeks, and remain at a low level for at least several additional weeks^17^,^18^,^19^. Considering dynamic and complex changes in cortisol levels caused by the cortisol surge and temporal entrainment of the adrenal clock at birth, we speculate that the foetal-type diurnal rhythm would be difficult to identify shortly after birth even though it is preserved.

We conducted a prospective study in a cohort of preterm and term infants to investigate whether a foetal-type adrenal rhythm is retained in newborn infants.

## Results

### Study population and subjects’ characteristics

A total of 130 samples were obtained from 65 cumulative newborn infants. Seven subjects were studied twice at an interval of 9.9 (2.0) days (Supplemental Fig. 1). Three samples from two newborn infants were of insufficient volume, and one newborn infant was diagnosed with a major chromosomal aberration with manifestation of highly abnormal spontaneous movements and muscle tone. Data from these three subjects were excluded from the analysis.

The final cohort comprised 62 cumulative newborn infants (31.7–39.5 weeks of corrected age and 1–92 days of postnatal age) who were hospitalized because of low birth weight (n = 55), maternal hyper- or hypothyroidism (n = 2), or maternal gestational diabetes mellitus (n = 5) (Table 1). Six newborn infants were extremely low birth weight. Antenatal glucocorticoids were administered in 29 newborn infants 45.4 (27.9) days before the study day, whereas postnatal glucocorticoid was prescribed in seven newborn infants 49.2 (27.0) days before the saliva collection.

### Diurnal difference in salivary cortisol levels

**Figure 1:**
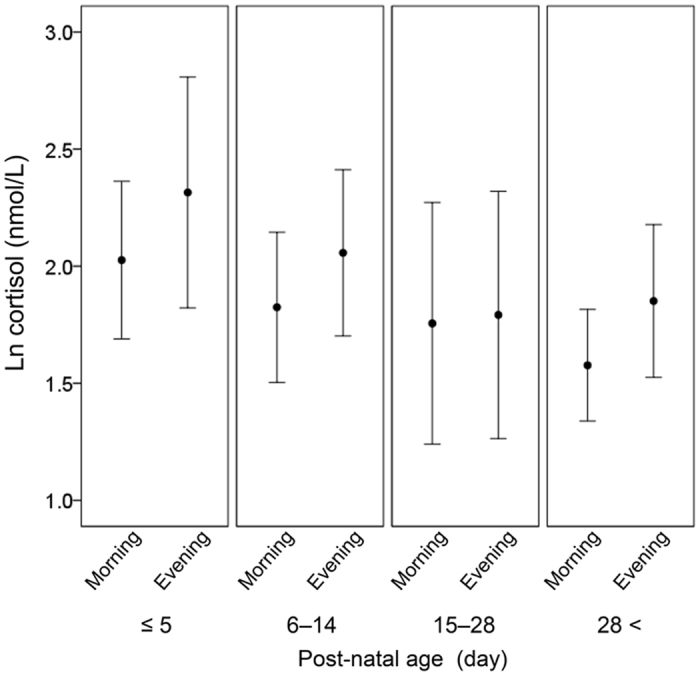
Diurnal and postnatal changes in salivary cortisol levels. Salivary cortisol levels were higher in the evening compared with those in the morning (p = 0.018). There was no interaction between the morning-evening difference in cortisol levels and gestational, postnatal and corrected age. Data are shown as mean (95% confidence interval).

Salivary cortisol levels were higher in the evening compared with those in the morning (p = 0.018), the difference of which was not associated with gestational age at birth and postnatal/corrected age at the time of sample collection. (Table 2A and Fig. 1).

### Control variables of salivary cortisol levels (adjusted for the diurnal difference

A persistent decline in cortisol levels with age was observed, with lower cortisol values between 15 and 28 days and at older than 28 days of life compared with those within 5 days of life (p = 0.040 and p = 0.003, respectively) (Table 2B; see also Supplemental Table 1 for analysis with additional variables). Lower cortisol levels were further associated with pregnancy-induced hypertension, very preterm birth <32 weeks gestation, preterm birth 32 ≤ <36 weeks gestation, and requirement for invasive/non-invasive mechanical ventilation after birth (p = 0.033, p < 0.001, p = 0.048, and p = 0.020, respectively). In contrast, higher cortisol levels were associated with vaginal delivery and dependence on non-invasive mechanical ventilation on the day of the study (p = 0.025 and p < 0.001, respectively). When these variables were re-assessed with adjustment for postnatal age in addition to the diurnal difference, pregnancy-induced hypertension, and dependence on non-invasive mechanical ventilation on the day of the study were still associated with cortisol levels (p = 0.002 and p < 0.001, respectively).

## Discussion

Thus far, diurnal adrenal rhythms entrained to a specific clock time have not been detected during the neonatal period. By recruiting a relatively large number of healthy preterm and term infants, we showed the presence of a cortisol secretion pattern that was dominant in the evening, and was observed from shortly after birth to at least approximately 2 months later. This diurnal pattern mimics the foetal adrenal rhythm, which is entrained in antiphase to the maternal rhythm^14^. Additionally, several clinically important independent variables of cortisol levels were identified, such as pregnancy-induced hypertension, delivery mode, gestational age and respiratory conditions, suggesting the impact of both intrinsic and extrinsic variables on the HPA axis of the neonates.

### Adrenal circadian rhythm in newborn infants

Studies in newborn infants have highlighted the presence of diurnal rhythms in body temperature and activity^20^,^21^. However, studies which serially assessed salivary cortisol levels supported the absence of diurnal rhythms entrained to clock times until the adult-type diurnal rhythm is established after 2–3 months of life^8^,^9^,^10^,^11^,^12^,^13^. In the current study, we observed a robust trend towards paradoxically higher salivary cortisol levels in the evening compared with those in the morning in newborn infants hospitalized at an intensive care unit. The mechanism of accelerated cortisol secretion in the evening is unknown. Given the presence of a foetal adrenal rhythm, which is entrained in antiphase to the maternal rhythm^14^, elevated cortisol levels in the evening observed in the current study might be the remainder of the foetal diurnal rhythm. However, extrinsic stimuli after birth may also cause diurnal cortisol rhythms in newborn infants either via the HPA axis or the autonomic system^22^,^23^. Future studies need to confirm the direct relationship between diurnal rhythms observed in the foetus and newborn infant using non-invasive biomarkers, which can be applied persistently before, during and after birth.

### Rationale for a “covert adrenal rhythm” in newborn infants

Although the concept of a preserved foetal-type adrenal rhythm after birth is theoretically relevant, studies have failed to capture this phenomenon except for subtle or temporary trends^15^,^24^ and observations based on a small fraction of the study population^12^,^19^. The HPA axis of the newborn infant is upregulated towards the time of delivery, leading to a surge in cortisol levels and then a gradual decline after birth^19^,^24^. During the surge, not only the absolute cortisol level, but its amplitude of temporal changes is also increased, in part due to an additional diurnal rhythm entrained at the time of birth^15^. In the current study, the difference in cortisol values between the morning and evening did not change with postnatal age, suggesting that the foetal-type and any other low-amplitude adrenal rhythms of cortisol would be difficult to identify shortly after birth with a limited number of subjects.

Consistent to previous reports, which demonstrated the function of the adrenal gland as a robust peripheral circadian clock^22^,^25^, prolonged preservation of a foetal-type adrenal rhythm was observed up to at least 2 months after birth. However, this finding contrasts from observations that an adult-type adrenal diurnal rhythm is generally identified from 2–3 months after birth in term infants^8^,^9^,^10^,^11^,^12^,^13^. For preterm infants, the transition from maternal and placental hormonal regulation to spontaneous regulation takes longer than that for term infants because of immature endocrine cells and their integration system^16^. Because our study cohort was mainly preterm infants, acquisition of the mature adrenal rhythm in the current study population might be substantially delayed. In addition to the intrinsic characteristics of preterm infants, extrinsic variables associated with care for preterm infants and sick term infants may contribute to persistent preservation of the foetal-type adrenal rhythm. While healthy term infants are exposed to day–night rhythms of lighting and other rhythms derived from feeding and family lifestyle, preterm infants are most often cared for at intensive care units, where the light cycle is dim and the feeding cycle is consistent throughout the day. A lack of input to the central circadian clock, in addition to immature function of the suprachiasmatic nucleus, may explain the delay in the maturational process of the peripheral clock in preterm infants.

### Independent variables of cortisol levels in newborn infants

Consistent to previous reports^26^,^27^,^28^,^29^, clinical variables, such as the delivery mode, gestational age, postnatal age, and respiratory distress, were associated with cortisol levels of neonates in our study cohort. Cortisol levels were lower in neonates who required invasive/non-invasive mechanical ventilation after birth, and were higher in neonates who were dependent on non-invasive mechanical ventilation on the day of the study. Associations between respiratory conditions and cortisol levels reported in previous studies are complex. When assessed shortly after birth, respiratory distress was associated with elevated cortisol levels in neonates between 31 and 36 weeks gestation^30^, whereas the presence of respiratory distress was associated with lower cortisol levels in very preterm infants^29^. Studies which serially monitored cortisol levels after birth suggested an altered impact of respiratory illness on cortisol secretion with postnatal age^26^. Taken together, the adrenal response to stress is likely to be affected by both gestational and postnatal age.

The impact of antenatal conditions on the postnatal adrenal function has not been fully investigated despite its clinical importance. Previous studies suggested postnatal upregulation of adrenal reactivity in association with antenatal stressors, such as maternal antepartum haemorrhage, depression and unfavourable socio-economic conditions^26^,^31^,^32^. In contrast, we found that pregnancy-induced hypertension was associated with lower cortisol levels in the current cohort. Considering that pregnancy-induced hypertension and subsequent placental dysfunction are known to increase risks for preterm birth, low-birth weight infants, intrauterine foetal death, and still birth, and that intrauterine conditions affect long-term endocrine regulation and incidence rates of endocrine and cardiovascular diseases^33^,^34^,^35^, further studies are required to reveal lifelong impacts of this clinical condition.

### Limitations of the study

Our study cohort was mainly preterm infants. We aimed to minimize or account for the influence of major known variables, such as feeding, painful procedures and invasive therapies^36^,^37^. However, we were unable to delineate whether an observed phenomenon was caused by intrinsic features characteristic to preterm infants or by extrinsic variables required for care of preterm infants. Our findings are based on salivary, but not plasma or serum, cortisol values. However, our previous study suggested that salivary cortisol levels were a reliable surrogate marker for plasma cortisol^1^. Unlike our previous study in which we collected eight saliva samples over 24 h^15^, we only assessed two samples from each participant in the current study. However, by recruiting a relatively large number of newborn infants, a consistent diurnal difference in salivary cortisol was observed, regardless of the postnatal age during the period of up to at least 2 months of birth. Cortisol assays used in previous studies had a relatively high cross-reactivity with cortisol analogues, such as cortisone and dehydroepiandrosterone^38^. However, the assay used in our previous and current studies has high specificity to cortisol, with cross-reactivities to cortisone and dehydroepiandrosterone less than 0.5%^39^.

## Conclusions

In neonates who were hospitalized at a neonatal intensive care unit, cortisol levels were higher in the evening than in the morning up to 8 weeks after birth. This dominant pattern of cortisol secretion in the evening suggests preservation of the foetal-type diurnal rhythm. Additionally, both intrinsic and extrinsic variables of cortisol levels were identified, such as maternal pregnancy-induced hypertension, delivery mode, gestational age and respiratory conditions after birth and at the time of the study. Whether these findings are consistently observed for healthy term infants needs to be investigated. The evolutional process of the cortisol rhythm from the foetal type to the adult type also needs to be investigated. Information on this process may help provide the optimal environment and developmental care for preterm and term infants to promote early acquisition of mature day–night rhythms.

## Materials and Methods

### Ethics approval and consent

This study was conducted in compliance with the Declaration of Helsinki and under the approval of the Ethics Committee of Kurume University School of Medicine. Written informed consent was obtained from a parent of each participating neonate.

### Study population

This study was conducted as part of a project, which aimed to delineate the control variables of adrenal function and its rhythm. Between January 2013 and June 2014, study days were assigned approximately twice a week on a calendar. Newborn infants between 30 and 40 weeks of corrected age, who were hospitalized at a tertiary neonatal intensive care unit (Kurume University Hospital, Kurume, Fukuoka, Japan), and were available for serial saliva collection on the study day, were recruited. Based on our previous data in preterm and term infants, which observed biphasic diurnal increase in cortisol in the late afternoon and in correspondence with the birth time^15^, a relatively large sample size of 61 was calculated to provide sufficient power to detect a significant difference in cortisol levels between the morning and evening. Newborn infants (i) who underwent phototherapy within 24 h of the study, (ii) who had not been weaned from intensive care, such as invasive mechanical ventilation and continuous intravenous infusion, (iii) who received glucocorticoid replacement therapy for the treatment of chronic lung disease or pressor-resistant hypotension within 1 week of the study, and (iv) whose oral or enteral feeding had not been established, were not included within the study cohort. This is because of the potential effect of treatments and procedural stress on cortisol levels. In our unit, we provided cycled lighting aimed at 100–200 lux during the day (0700 to earlier than 1900 h) and 10–30 lux during the night (1900 to earlier than 700 h). All newborn infants within the study population were cared for within a closed incubator, which was covered by a quilt cover.

### Sample collection and assay

Saliva samples were collected before and 1 h after regular feeding at 10:00 and 19:00 h. For sample collection, an absorbent swab (SalivaBio; Salimetrics LLC, State College, PA, USA) was gently inserted into the newborn infant’s mouth for approximately 5 min, allowing the swab end to absorb sufficient saliva. The sample was immediately centrifuged at 3000 rpm. Samples were initially kept at 4 °C, and were frozen at −80 °C after the last sample for the newborn infant was collected. Levels of salivary cortisol were determined by enzyme immunoassay (high-sensitivity salivary cortisol ELISA kit; Salimetrics LLC). The limit of detection of this assay in our laboratory was 0.19 nmol/L, and the intra- and inter-assay coefficients of variation were 5.43% and 6.41%, respectively. Data collection was repeated up to twice for each patient with a minimum interval of 7 days.

### Clinical background variables

Clinical data were collected from an electronic patient record, including information (i) before delivery (maternal glucocorticoid administration, chorioamnionitis, pregnancy-induced hypertension, multiple births, intravenous tocolysis, maternal hospital stay until delivery, and delivery mode), (ii) after birth (gestational age, sex, Apgar scores, intrauterine growth restriction, and requirement for invasive/non-invasive mechanical ventilation), and (iii) at the time of the study (postnatal age, feeding mode, clinical record of postnatal glucocorticoid administration, requirement for non-invasive mechanical ventilation using a nasal continuous positive airway pressure device, and invasive blood sampling in the morning of the study).

### Data analysis

Values are presented as mean (standard deviation) unless otherwise specified. For the current study, only cortisol values before feeding were analysed. Salivary cortisol values were normalized by transforming data into natural logarithms. Clinical variables were dichotomized using clinically relevant thresholds. Generalized estimating equations were used to simultaneously assess (i) the diurnal difference in cortisol values and its interaction with postnatal age, and (ii) the dependence of salivary cortisol values on 10 selected clinical variables by incorporating repeated observations in the morning and evening. P-values from multiple comparisons were presented without correction as clinical variables were chosen restrictively based on a priori hypothesis. Because of a known age-dependent decline in cortisol levels after birth^17^,^18^,^19^, independent variables were re-analysed with correction for postnatal age.

## Additional Information

**How to cite this article**: Kinoshita, M. *et al*. Paradoxical diurnal cortisol changes in neonates suggesting preservation of foetal adrenal rhythms. *Sci. Rep.* **6**, 35553; doi: 10.1038/srep35553 (2016).

## Supplementary Material

Supplementary Information

## Figures and Tables

**Table 1:** Background clinical variables.

Variables	
Female/male	35/27
Multiple births	15
Birth weight (g)	1671 ± 579
Intrauterine growth restriction	20
Vaginal delivery	26
Caesarean section	36
Gestational age (weeks)	32.3 ± 3.7
Postnatal age (days)	24 ± 23
Corrected age (weeks)	35.8 ± 1.3
Body weight on the day of saliva collection (g)	1990 ± 337
Water quotient* (ml/kg/d)	145 ± 14
Use of glucocorticoid	
Antenatal	29
Postnatal	7

^*^The water quotient is calculated by the fluid intake per 24 h divided by the body weight.

Values are shown as the number or mean ± standard deviation.

**Table 2:** Dependence of salivary cortisol levels on internal and external variables.

Variable	n	Ln cortisol (nmol/L)	β		p	
			Mean	95% CI		
**A: Internal variable (diurnal difference)**						
Morning	62	1.76 (0.62)	0.799	(0.664, 0.962)	0.018	
Evening	62	1.99 (0.74)	Reference			

**B: External variables**						
**Variables**	**n**	**Ln cortisol (nmol/L)**	**β**		**p**	
			**Mean**	**95% CI**	**Unadjusted**	**Adjusted**
**Antenatal variables**						
Pregnancy-induced hypertension						
Yes	10	1.61 (0.63)	0.731	(0.547, 0.975)	0.033	0.002
No	52	1.92 (0.69)	Reference			
Antenatal glucocorticoid						
Yes	29	1.77 (0.62)	0.815	(0.621, 1.071)	0.142	0.794
No	33	1.97 (0.74)	Reference			
**Postnatal variables**						
Delivery mode						
Vaginal	26	2.06 (0.70)	1.377	(1.041, 1.823)	0.025	0.087
Caesarean	36	1.74 (0.65)	Reference			
Gestational age (week)						
<32	23	1.66 (0.56)	0.543	(0.389, 0.758)	<0.001	0.083
32 ≤ <36	31	1.93 (0.76)	0.709	(0.504, 0.997)	0.048	
36≤	8	2.27 (0.57)	Reference			
Apgar score (5 min.)						
<7	8	1.65 (0.59)	0.777	(0.564, 1.069)	0.121	0.467
7≤	54	1.91 (0.70)	Reference			
Need for invasive/non-invasive mechanical ventilation						
Yes	31	1.71 (0.60)	0.725	(0.553, 0.950)	0.020	0.391
No	31	2.04 (0.75)	Reference			
Postnatal glucocorticoid						
Yes	7	1.67 (0.62)	0.793	(0.508, 1.237)	0.306	0.757
No	55	1.90 (0.70)	Reference			
**Variables on the day of study**						
Age (day)						
≤5	10	2.17 (0.59)	Reference			NA
5 < ≤14	21	1.94 (0.74)	0.795	(0.562, 1.124)	0.194	
15 < ≤28	10	1.77 (0.71)	0.673	(0.461, 0.982)	0.040	
28<	21	1.71 (0.64)	0.634	(0.470, 0.855)	0.003	
Blood sampling						
Yes	19	2.08 (0.76)	1.341	(0.981, 1.834)	0.066	0.457
No	43	1.78 (0.64)	Reference			
Need for non-invasive mechanical ventilation						
Yes	3	2.34 (0.24)	1.635	(1.271, 2.104)	<0.001	<0.001
No	59	1.85 (0.70)	Reference			

Abbreviations: β, standardized coefficient. CI, confidence interval. NA, not applicable.

P-values are presented with and without correction for postnatal age.

## References

[b1] Okamura H. . Noninvasive surrogate markers for plasma cortisol in newborn infants: utility of urine and saliva samples and caution for venipuncture blood samples. The Journal of clinical endocrinology and metabolism 99, E2020–E2024, 10.1210/jc.2014-2009 (2014). 25078034

[b2] Voegtline K. M. & Granger D. A. Dispatches from the interface of salivary bioscience and neonatal research. Frontiers in endocrinology 5, 25, 10.3389/fendo.2014.00025 (2014). 24624119 PMC3940893

[b3] Walker R. F., Riad-Fahmy D. & Read G. F. Adrenal status assessed by direct radioimmunoassay of cortisol in whole saliva or parotid saliva. Clinical chemistry 24, 1460–1463 (1978). 688602

[b4] Umeda T. . Use of saliva for monitoring unbound free cortisol levels in serum. Clinica chimica acta; international journal of clinical chemistry 110, 245–253 (1981). 6261989 10.1016/0009-8981(81)90353-3

[b5] Kirschbaum C. & Hellhammer D. H. Salivary cortisol in psychoneuroendocrine research: recent developments and applications. Psychoneuroendocrinology 19, 313–333 (1994). 8047637 10.1016/0306-4530(94)90013-2

[b6] Dockray S. & Steptoe A. Chronotype and diurnal cortisol profile in working women: differences between work and leisure days. Psychoneuroendocrinology 36, 649–655, 10.1016/j.psyneuen.2010.09.008 (2011). 20950941

[b7] Shirtcliff E. A., Granger D. A., Schwartz E. & Curran M. J. Use of salivary biomarkers in biobehavioral research: cotton-based sample collection methods can interfere with salivary immunoassay results. Psychoneuroendocrinology 26, 165–173 (2001). 11087962 10.1016/s0306-4530(00)00042-1

[b8] Price D. A., Close G. C. & Fielding B. A. Age of appearance of circadian rhythm in salivary cortisol values in infancy. Archives of disease in childhood 58, 454–456 (1983). 6859940 10.1136/adc.58.6.454 PMC1628010

[b9] de Weerth C., Zijl R. H. & Buitelaar J. K. Development of cortisol circadian rhythm in infancy. Early human development 73, 39–52 (2003). 12932892 10.1016/s0378-3782(03)00074-4

[b10] Vermes I., Dohanics J., Toth G. & Pongracz J. Maturation of the circadian rhythm of the adrenocortical functions in human neonates and infants. Hormone research 12, 237–244 (1980). 7399396 10.1159/000179126

[b11] Santiago L. B., Jorge S. M. & Moreira A. C. Longitudinal evaluation of the development of salivary cortisol circadian rhythm in infancy. Clinical endocrinology 44, 157–161 (1996). 8849569 10.1046/j.1365-2265.1996.645466.x

[b12] Antonini S. R., Jorge S. M. & Moreira A. C. The emergence of salivary cortisol circadian rhythm and its relationship to sleep activity in preterm infants. Clinical endocrinology 52, 423–426 (2000). 10762284

[b13] Spangler G. The emergence of adrenocortical circadian function in newborns and infants and its relationship to sleep, feeding and maternal adrenocortical activity. Early human development 25, 197–208 (1991). 1935741 10.1016/0378-3782(91)90116-k

[b14] Seron-Ferre M., Riffo R., Valenzuela G. J. & Germain A. M. Twenty-four-hour pattern of cortisol in the human fetus at term. American journal of obstetrics and gynecology 184, 1278–1283 (2001). 11349202 10.1067/mob.2001.113322

[b15] Iwata O. . Diurnal cortisol changes in newborn infants suggesting entrainment of peripheral circadian clock in utero and at birth. The Journal of clinical endocrinology and metabolism 98, E25–E32, 10.1210/jc.2012-2750 (2013). 23150686

[b16] Bolt R. J., van Weissenbruch M. M., Lafeber H. N. & Delemarre-van de Waal H. A. Development of the hypothalamic-pituitary-adrenal axis in the fetus and preterm infant. Journal of pediatric endocrinology & metabolism: JPEM 15, 759–769 (2002). 12099385 10.1515/jpem.2002.15.6.759

[b17] Wittekind C. A., Arnold J. D., Leslie G. I., Luttrell B. & Jones M. P. Longitudinal study of plasma ACTH and cortisol in very low birth weight infants in the first 8 weeks of life. Early human development 33, 191–200 (1993). 8223315 10.1016/0378-3782(93)90145-k

[b18] Arnold J. . Longitudinal study of plasma cortisol and 17-hydroxyprogesterone in very-low-birth-weight infants during the first 16 weeks of life. Biology of the neonate 72, 148–155 (1997). 9303213 10.1159/000244478

[b19] Kidd S., Midgley P., Nicol M., Smith J. & McIntosh N. Lack of adult-type salivary cortisol circadian rhythm in hospitalized preterm infants. Hormone research 64, 20–27, 10.1159/000087324 (2005). 16088204

[b20] Nishihara K., Horiuchi S., Eto H. & Uchida S. The development of infants’ circadian rest-activity rhythm and mothers’ rhythm. Physiology & behavior 77, 91–98 (2002). 12213506 10.1016/s0031-9384(02)00846-6

[b21] Batinga H. . Ontogeny and aging of the distal skin temperature rhythm in humans. Age (Dordrecht, Netherlands) 37, 29, 10.1007/s11357-015-9768-y (2015). PMC4375132 25813804

[b22] Gamble K. L., Berry R., Frank S. J. & Young M. E. Circadian clock control of endocrine factors. Nature reviews. Endocrinology 10, 466–475, 10.1038/nrendo.2014.78 (2014). PMC4304769 24863387

[b23] Ishida A. . Light activates the adrenal gland: timing of gene expression and glucocorticoid release. Cell metabolism 2, 297–307, 10.1016/j.cmet.2005.09.009 (2005). 16271530

[b24] Dorn F. . Influence of acoustic stimulation on the circadian and ultradian rhythm of premature infants. Chronobiology international 31, 1062–1074, 10.3109/07420528.2014.948183 (2014). 25133792

[b25] Tsang A. H., Astiz M., Friedrichs M. & Oster H. Endocrine regulation of circadian physiology. The Journal of endocrinology 230, R1–R11, 10.1530/JOE-16-0051 (2016). 27106109

[b26] Ng P. C. . Reference ranges and factors affecting the human corticotropin-releasing hormone test in preterm, very low birth weight infants. The Journal of clinical endocrinology and metabolism 87, 4621–4628, 10.1210/jc.2001-011620 (2002). 12364445

[b27] Bird J. A., Spencer J. A., Mould T. & Symonds M. E. Endocrine and metabolic adaptation following caesarean section or vaginal delivery. Archives of disease in childhood. Fetal and neonatal edition 74, F132–F134 (1996). 8777662 10.1136/fn.74.2.f132 PMC2528519

[b28] Bagnoli F. . ACTH and cortisol cord plasma concentrations in preterm and term infants. Journal of perinatology: official journal of the California Perinatal Association 33, 520–524, 10.1038/jp.2012.165 (2013). 23306940

[b29] Scott S. M. & Watterberg K. L. Effect of gestational age, postnatal age, and illness on plasma cortisol concentrations in premature infants. Pediatric research 37, 112–116, 10.1203/00006450-199501000-00021 (1995). 7700725

[b30] Gunes T., Koklu E., Ozturk M. A., Koklu S. & Cetin N. Evaluation of serum cortisol levels in a relatively large and mature group of ventilated and nonventilated preterm infants with respiratory distress syndrome. American journal of perinatology 23, 335–339, 10.1055/s-2006-948222 (2006). 16841278

[b31] Luecken L. J. . Prenatal stress, partner support, and infant cortisol reactivity in low-income Mexican American families. Psychoneuroendocrinology 38, 3092–3101, 10.1016/j.psyneuen.2013.09.006 (2013). 24090585 PMC3844006

[b32] Fernandes M., Stein A., Srinivasan K., Menezes G. & Ramchandani P. G. Foetal exposure to maternal depression predicts cortisol responses in infants: findings from rural South India. Child: care, health and development 41, 677–686, 10.1111/cch.12186 (2015). 25131942

[b33] Baschat A. A. . Predictors of neonatal outcome in early-onset placental dysfunction. Obstetrics and gynecology 109, 253–261, 10.1097/01.aog.0000253215.79121.75 (2007). 17267821

[b34] Seckl J. R. Physiologic programming of the fetus. Clinics in perinatology 25, 939–962, vii (1998). 9891623

[b35] Chen Y. C., Sheen J. M., Tiao M. M., Tain Y. L. & Huang L. T. Roles of melatonin in fetal programming in compromised pregnancies. International journal of molecular sciences 14, 5380–5401, 10.3390/ijms14035380 (2013). 23466884 PMC3634509

[b36] Kleberg A. . Lower stress responses after Newborn Individualized Developmental Care and Assessment Program care during eye screening examinations for retinopathy of prematurity: a randomized study. Pediatrics 121, e1267–e1278, 10.1542/peds.2006-2510 (2008). 18450869

[b37] Gibson E. L. . Increased salivary cortisol reliably induced by a protein-rich midday meal. Psychosomatic medicine 61, 214–224 (1999). 10204975 10.1097/00006842-199903000-00014

[b38] Inder W. J., Dimeski G. & Russell A. Measurement of salivary cortisol in 2012 - laboratory techniques and clinical indications. Clinical endocrinology 77, 645–651, 10.1111/j.1365-2265.2012.04508.x (2012). 22812714

[b39] *Salimetrics LLC. Expanded Range High Sensitivity Salivary Cortisol Enzyme Immunoassay Kit.* https://www.salimetrics.com/assets/documents/1-3002.pdf. (Last accessed on 07/09/2016).

