# Supplementary material for: Paradoxical diurnal cortisol changes in neonates suggesting preservation of foetal adrenal rhythms
Source: Sci Rep. 2016 Oct 18;6:35553. doi: 10.1038/srep35553 (PMC5067652; doi:10.1038/srep35553)
Supplement: Supplementary Information [file srep35553-s1.doc]

**Title: Paradoxical diurnal cortisol changes in neonates suggesting preservation of foetal adrenal rhythms**

**Authors:**

Masahiro Kinoshita, MD^1^, Sachiko Iwata, MD, PhD^1,2^, Hisayoshi Okamura, PhD^2^, Mamoru Saikusa, MD^1^, Naoko Hara, MD^1^, Chihoko Urata^1^, Yuko Araki, PhD^3^, Osuke Iwata, MD, PhD^1,2^

**Affiliations:**

1. Department of Paediatrics and Child Health, Kurume University School of Medicine, Fukuoka, Japan

2. Centre for Developmental and Cognitive Neuroscience, Kurume University School of Medicine, Fukuoka, Japan

3. Faculty of Informatics, Shizuoka University, 836, Ohya, Suruga-ku, Shizuoka, Japan

**Correspondence to:**

Dr Osuke Iwata

Centre for Developmental and Cognitive Neuroscience, Kurume University School of Medicine

67 Asahimachi, Kurume, Fukuoka, 830-0011 Japan.

E-mail: o.iwata@ucl.ac.uk Tel: +81 942 31-7565 Fax: +81 942 38-1792

Online supplemental files:

Online supplemental figure 1. Distribution of gestational age at birth and postnatal and corrected age on the day of the study.


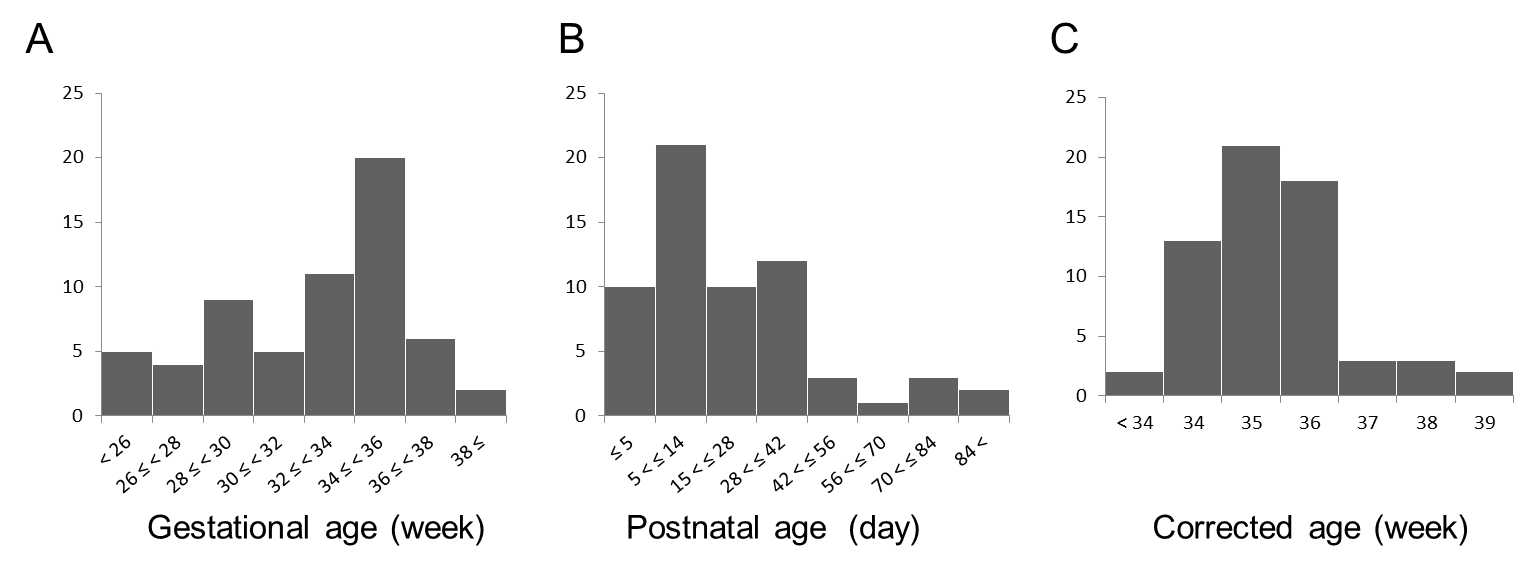


Online supplemental table 1. Dependence of salivary cortisol levels on external variables

| **Variables** | | | n | Ln cortisol (nmol/L) | β | | p | |
| --- | --- | --- | --- | --- | --- | --- | --- | --- |
|  |  |  |  |  | Mean | 95% CI | Unadjusted | Adjusted |
| **Antenatal variables** | | |  |  |  |  |  |  |
|  | Intrauterine growth restriction | | |  |  |  |  |  |
|  |  | Yes | 20 | 1.92 (0.76) | 1.076 | (0.769, 1.505) | 0.668 | 0.921 |
|  |  | No | 42 | 1.85 (0.66) | Reference | |  |  |
|  | Multiple birth | |  |  |  |  |  |  |
|  |  | Yes | 15 | 1.81 (0.73) | 0.925 | (0.647, 1.321) | 0.668 | 0.782 |
|  |  | No | 47 | 1.89 (0.68) | Reference | |  |  |
|  | Intravenous tocolysis | |  |  |  |  |  |  |
|  |  | Yes | 41 | 1.86 (0.73) | 0.965 | (0.737, 1.263) | 0.794 | 0.485 |
|  |  | No | 21 | 1.90 (0.62) | Reference | |  |  |
|  | Maternal hospitalisation before delivery　(d) | | | | |  |  |  |
|  |  | < 10 | 38 | 1.86 (0.66) | Reference | |  |  |
|  |  | ≥ 10 | 24 | 1.90 (0.74) | 1.039 | (0.760, 1.420) | 0.813 | 0.472 |
|  | Chorioamnionitis | |  |  |  |  |  |  |
|  |  | Yes | 18 | 1.93 (0.59) | 1.082 | (0.820, 1.428) | 0.576 | 0.343 |
|  |  | No | 44 | 1.85 (0.73) | Reference | |  |  |
| **Postnatal variables** | | |  |  |  |  |  |  |
|  | Sex |  |  |  |  |  |  |  |
|  |  | Male | 27 | 1.88 (0.73) | 1.007 | (0.756, 1.341) | 0.961 | 0.753 |
|  |  | Female | 35 | 1.87 (0.67) | Reference | |  |  |
|  | Apgar score (1 min.) | |  |  |  |  |  |  |
|  |  | < 7 | 22 | 1.81 (0.65) | 0.909 | (0.688, 1.201) | 0.503 | 0.509 |
|  |  | 7 ≤ | 40 | 1.91 (0.72) | Reference | |  |  |
|  | Need for tracheal intubation | | | | | |  |  |
|  |  | Yes | 18 | 1.80 (0.64) | 0.9 | (0.673, 1.204) | 0.478 | 0.339 |
|  |  | No | 44 | 1.90 (0.71) | Reference | |  |  |
|  | Hypothyroidism | |  |  |  |  |  |  |
|  |  | Yes | 3 | 2.16 (0.72) | 1.344 | (1.086, 1.663) | 0.007 | <0.001 |
|  |  | No | 59 | 1.86 (0.69) | Reference | |  |  |
|  | Chronic lung disease | |  |  |  |  |  |  |
|  |  | Yes | 15 | 1.68 (0.60) | 0.77 | (0.561, 1.057) | 0.106 | 0.596 |
|  |  | No | 47 | 1.94 (0.71) | Reference | |  |  |

Abbreviations: β, standardised coefficient. CI, confidence interval. NA, not applicable.

P-values are presented with or without adjusted for post-natal age.
